# The cardio-metabolic impact of taking commonly prescribed analgesic drugs in 133,401 UK Biobank participants

**DOI:** 10.1371/journal.pone.0187982

**Published:** 2017-12-06

**Authors:** Sophie Cassidy, Michael I. Trenell, Kirstie N. Anderson

**Affiliations:** 1 Faculty of Medical Sciences, Institute of Cellular Medicine, Newcastle University, Newcastle upon Tyne, United Kingdom; 2 Institute of Neuroscience, Newcastle University, Newcastle upon Tyne, United Kingdom; Tokai Daigaku, JAPAN

## Abstract

**Objective:**

There has been a significant increase in the prescribing of medication for chronic non-cancer pain. In a UK population sample, we aimed to assess cardio-metabolic (CM) health in those taking these chronic pain medications.

**Methods:**

133,401 participants from the UK Biobank cohort were studied. BMI, waist cm and hypertension were compared between those on drugs prescribed for chronic pain *and* CM drugs to those on CM drugs only. Multiple confounders were controlled for.

**Results:**

Those taking opiates *and* CM drugs had the worst CM health profile with a 95%, 82% and 63% increased odds of reporting obesity, ‘very high risk’ waist circumference and hypertension, respectively (OR [95% CI] 1.95 [1.75–2.17], 1.82 [1.63–2.03], 1.63 [1.45–1.84]), compared to those on CM drugs alone. Those taking neuropathic pain medications *and* CM drugs also demonstrate worse CM profile than those taking CM drugs only.

**Conclusions:**

The impact of medications for chronic pain and sleep upon CM health and obesity is of concern for these classes of drugs which have been recently labelled as dependency forming medications. The results from this cross sectional study warrants further investigation and adds further support to calls for these medications to be prescribed for shorter periods.

## Introduction

Obesity, cardiovascular disease and type 2 diabetes represent significant personal, societal and economic burden [1,2]. The inter-relationship between metabolic and cardiovascular disease is termed cardio-metabolic (CM) health. Those with both CVD and type 2 diabetes have a particularly poor prognosis and require aggressive risk factor intervention [3]. Obesity now affects 34.9% of all US adults and 26% of UK adults with a greater than doubling of the prevalence over the last 30 years [4].

There is increasing recognition of the metabolic impact of many prescribed analgesic medications used for relief of chronic pain. Certain medications have been used far more widely in recent years, in particular those for chronic non cancer pain. This includes opioids, the alpha2delta ligands pregabilin and gabapentin and certain antidepressants shown to decrease neuropathic pain, in particular amitriptyline and more recently duloxetine which is specifically licensed for diabetic neuropathic pain. Many of these medications have been labelled as dependence-forming medications (DFM) whereby patients require continual use to maintain a state of normality and avoid symptoms of withdrawal [5]. Prescribing data shows a substantial rise in these prescriptions over the last ten years in the UK and the US. An estimated 3–4% of US adults are prescribed opioids [6]. The use of strong opioids has increased alongside gabapentin and pregabilin [7].

The increased use of opioid medication has led to increasing recognition of their side effects when used chronically and the use of long term opioid medication has become increasingly controversial with recent, far reaching changes to reduce prescribing in the US [8]. The dangers of addiction, sleep disordered breathing, daytime sedation and accidental overdose are well known and researched [9]. The impact of opioids upon appetite, metabolism and the insulin axis is less well studied [10]. More recently cohort studies have suggested that those using chronic opiate prescriptions in particular have an increased mortality rate from all causes but there may be many confounding factors when studying those with chronic pain and the reasons for this association remain debated [11].

Weight gain with neuropathic pain meds is described but often in small studies looking at the impact upon the first 6–12 weeks of prescription [12,13]. There have been few large studies addressing the potential association between commonly prescribed analgesic drugs and CM health. However, smaller studies following patients prescribed gabapentin, pregabilin, amitriptyline have shown increased BMI in those followed up between periods of 3–6 months. Weight gain is cited as one of the commonest reasons for discontinuing these medications [14]. Opiates have been shown to cause insulin resistance but whether they clearly cause weight gain over time remains debated [10]. Many of the analgesic drugs cause sedation and increase the risk of sleep disordered breathing. Sleep disturbance in itself causes insulin resistance with increasing recognition of the association between sleep and circadian rhythm disturbance, worse CM health and increased obesity [15].

The UK Biobank is a large population-based cohort which allows measurement of commonly used prescription drugs alongside important indicators of CM health, physical activity and lifestyle behaviours [16]. Our primary aim was to assess CM health in those taking commonly prescribed analgesic drugs. Our secondary objective was to further explore associations with lifestyle factors such as physical activity, diet and sleep duration to try and understand the mechanism of any metabolic disturbance.

Given that many participants are prescribed combinations of opiates and neuropathic pain relief, we elected to study those on any combination of prescribed analgesic medication including opiates, antidepressants recommended for chronic pain and the alpha2delta ligands pregabilin and gabapentin. We hypothesized that this group would be more likely to have poor CM health and increased obesity and that this association would persist as an independent risk factor when controlling for other lifestyle factors.

## Methods

### Population and study design

A cross-sectional analysis was conducted on baseline data from the UK Biobank. The UK Biobank is a large, population-based cohort study examining the interrelationships between environment, lifestyle and genes [16]. Around 9.2 million invitations were mailed to recruit 502,664 adults (response rate 5.5%) aged between 37 and 73 years. Between 2007–2010 participants attended a baseline assessment at one of the 22 centres located across the UK. During an assessment centre visit, there were six stages; consent, touchscreen questionnaire, verbal interview, eye measures, physical measures and blood/urine sample collection. Details of procedures have been previously published [16]. Participant written informed consent was obtained prior to data collection. All data extracted were deidentified for analysis.

### Baseline measurements

Sociodemographic, smoking, alcohol, physical activity, dietary intake and sleep duration data were collected from the touchscreen questionnaire. Smoking and alcohol status were obtained by asking participants to respond to ‘Prefer not to answer’, ‘Never’, ‘Previous’ and ‘Current’. Physical activity was assessed using adapted questions from the validated short International Physical Activity Questionnaire (IPAQ)[17] which collects information on the frequency and duration of time spent in walking, moderate and vigorous activity in the previous 7 days. Data processing rules for IPAQ were followed [18]. According to the current UK physical activity guidelines [19] we identified those who reached 150 minutes of moderate or 75 minutes of vigorous activity per week, or a combination of both. Walking was considered as moderate activity. Participants reported how long they spent watching television (TV) on a typical day. This was asked twice to those who responded >8 hours, therefore high values were deemed robust. Those who reported more than 3 hours/day were identified based upon previous literature [20]. Diet was reported using the Food Frequency Questionnaire [21] and healthy eating was assessed using the UK’s fruit and vegetable guidelines of 5 portions per day [21]. The townsend deprivation index combines information on housing, employment, car availability and social class, with higher values representing lower socioeconomic status. This was calculated before participants joined the UK Biobank and was based on the preceding national census data, with each participant assigned a score corresponding to the home postcode [16].

To measure sleep duration, participants were asked ‘About how many hours sleep do you get in every 24 hours?’ (please include naps). This was asked twice to those who responded >12 hours. As sleep shows a ‘U’ shaped relationship with CM risk [22], we created 3 sleep duration groups of <6 (poor sleep), 6–9 (good sleep), and >9 hours (poor sleep) per night.

Body mass index (BMI) was calculated from: weight (kg)/height(m)^2^. Weight was measured using the Tanita BC-418MA body composition analyser, to the nearest 0.1 kg and height was measured using a Seca 202 height measure. Waist circumference (cm) was measured at the level of the umbilicus using a Wessex non-stretchable sprung tape measure, which has previously been adopted in large health studies [23]. Participants were asked to adjust clothing for accuracy, and all staff were trained in taking these measures. Self-report disease status (including Sleep Apnoea and hypertension) and medication use was obtained from participants during the touchscreen questionnaire, which was then entered and verified by a UK Biobank nurse after further questioning during the verbal interview. The interview provided extra information such as time of diagnosis, and if the trained nurse decides that the illness or medication had been incorrectly selected, they could remove the selection.

Four groups were created depending on self-reported medication use. These included:

1. CM controls: those taking the most commonly prescribed CM drugs according to the UK prescribing data available from 2010 (http://content.digital.nhs.uk/gpprescribingdata). This included atenolol, ramipril, bendroflumethazide, losartan, clopidogrel, simvastatin, and atorvastatin (the exact list from the UK biobank coding can be seen in S1 Table).
2. Neuropathic pain medication which was defined as; Pregabilin (Lyrica), Gabapentin (Neurontin), Amitriptyline, Duloxetine (Cymbalta), Amitriptyline + chlordiazepoxide
3. Opiate medication, those who reported taking any of the following prescribed medications; Morphine sulphate tablets (MST), Tramadol, Paracetomol + Tramadol, oramorph (9 different doses), Cocodamol, codydramol (paracetamol + dihydrocodeine), Fentanyl patch, Buprenorphine
4. Combination prescriptions that included those prescribed both neuropathic pain medication and opiates

### Statistical analysis

Socio-demographics and lifestyle behaviours were compared across medication groups, but owing to the large sample size, Pearson’s χ2 deemed any small difference in group proportions as significant, therefore, these results are not reported. Individuals with missing data on either BMI, waist cm, or hypertension (main outcomes) were excluded. Their socio-demographics, which were similar to the main cohort, but included a higher proportion of males and higher deprivation scores, can be seen in S2 Table. Binary logistic regression was used to determine the odds of being obese, having a ‘very high risk’ waist cm, or hypertensive, across medication groups. Adjusted ORs, with 95% CIs were reported. All logistic regression models were adjusted for: age (reference = ‘37–49’); gender (reference = ‘Female’); townsend Deprivation Index (reference = ‘least deprived); ethnicity (reference = ‘White/British); alcohol (reference = ‘Never’); smoking (reference = ‘Never’); meets fruit/vegetable guidelines (reference = ‘YES’); meets physical activity guidelines (reference = ‘YES’) and sleep duration (reference = good sleep). Of the 133,401 cohort, data was missing for; Townsend Deprivation Index (0.1%), ethnicity (0.5%), smoking status (0.6%), alcohol status (0.2%), physical activity (21.4%), and fruit and vegetable guidelines (3.6%), sleep (1.0%) therefore, these cases were excluded from the logistic regression models and the final model was *n = 102*,*100*. All statistical analysis was performed using SPSS, V21.0 (IBM, Armonk, New York, USA) and significance was set at p<0.05, two sided.

## Results

Of the 502 664 UK Biobank participants, excluding those with missing data for BMI, waist cm, or hypertension, 125,978 (25.2%) were taking CM drugs and 7,423 (1.5%) were taking some form of prescribed analgesic alongside CM drugs, their demographics are shown in Table 1. 4461 were prescribed neuropathic pain medication, 2125 using opiates and 837 using both neuropathic pain drugs and opiates, in addition to their CM drugs. There were a higher proportion of females over 60 years old taking prescribed analgesics and were more deprived than the CM control group (Table 1).

**Table 1:** Socio-demographics of each medication group.

	% within each disease group			
	CM controls *(125*,*978)*	Neuropathic pain meds *(4461)*	Opiates *(2125)*	Neuropathic pain meds + Opiates *(837)*
SOCIO-DEMOGRAPHICS				
**% Male**	56.0	39.7	48.4	39.7
**Age *(n)***	*125*,*978*	*4461*	*2125*	*837*
**Age (mean±SD)**	61±7	61±6	60±7	58±7
**Age Groups**				
37–49	8.4	7.8	8.2	11.0
50–59	27.0	28.4	28.8	35.7
60–73	64.6	63.8	62.9	53.3
**Townsend deprivation quintile *(n)***	*125*,*829*	*4458*	*2122*	*834*
1 (least deprived)	19.2	15.3	11.7	9.6
2	19.6	16.9	13.9	14.4
3	19.9	18.4	16.4	15.1
4	19.7	20.0	19.8	21.5
5 (most deprived)	21.5	29.5	38.2	39.4
**Ethnicity *(n)***	*125*,*341*	*4439*	*2113*	*833*
White/British	94.4	95.0	94.7	95.3
Mixed	0.5	0.5	0.4	0.4
Asian	2.4	2.1	2.1	2.3
Black African	1.7	1.5	1.8	1.0
Chinese	0.2	0.0	0.1	0.0
Other	0.9	0.9	0.9	1.1

There was a higher proportion of current smokers in those taking analgesics, but a lower proportion of current drinkers, compared to those on CM drugs only. The proportion of adults sleeping less than 6 hours or more than 9 hours per night was higher in the analgesic groups. Those taking combination prescriptions of both neuropathic pain medication and opiates showed the worst sleep patterns compared to CM controls (Good sleep (6-9hrs): 71.4% *vs* 91.3%, respectively). Those on analgesics reported lower physical activity levels, and greater TV viewing but did not show worse eating behaviours compared to the CM controls (Table 2).

**Table 2:** Cardio-metabolic health and lifestyle characteristics of medication groups.

	% within each disease group			
	CM controls *(125*,*978)*	Neuropathic pain meds *(4461)*	Opiates *(2125)*	Neuropathic pain meds + Opiates *(835)*
**Cardio-metabolic health**				
**BMI**	*125*,*978*	*4461*	*2125*	*837*
<18.5 (underweight)	0.2	0.3	0.3	0.5
18.5–24.9 (normal weight)	20.4	15.7	11.2	9.3
25–29.9 (overweight)	44.0	36.6	32.8	31.9
≥30 (obese)	35.4	47.4	55.6	58.3
**Waist cm groups (MALES) (n)**	*70*,*602*	*1770*	*1028*	*332*
<94 (low risk)	28.8	19.1	17.0	13.3
94–102 (high risk)	32.9	28.9	27.2	28.0
>102 (very high risk)	38.3	52.0	55.7	58.7
**Waist cm groups (FEMALES) *(n)***	*55*,*376*	*2691*	*1097*	*505*
<80 (low risk)	23.5	15.9	11.1	9.3
80–88 (high risk)	26.8	21.1	18.4	18.8
>88 (very high risk)	49.7	63.0	70.5	71.9
**Hypertension**	*125*,*978*	*4461*	*2125*	*837*
Report hypertension or essential hypertension	62.4	67.8	74.5	70.7
**Lifestyle**				
**Smoking**	*125*,*845*	*4454*	*2124*	*837*
Never	48.0	43.5	36.1	37.4
Previous	41.3	41.9	45.1	41.1
Current	10.2	13.8	17.9	20.4
Prefer not to answer	0.5	0.7	0.9	1.1
**Alcohol**	*125*,*845*	*4454*	*2124*	*837*
Never	5.1	8.3	8.5	10.0
Previous	4.1	10.4	11.1	19.1
Current	90.6	81.2	80.1	70.8
Prefer not to answer	0.1	0.0	0.2	0.0
**Sleep duration (hours/night)**	*124*,*803*	*4363*	*2084*	*816*
<6 (Poor sleep)	6.1	9.9	16.4	18.3
6–9 (Good sleep)	91.3	83.6	77.5	71.4
>9 (Poor sleep)	2.6	6.5	6.1	10.3
**Meets UK government physical activity guidelines** ^a^	*99*,*409*	*3289*	*1553*	*589*
NO	19.7	30.9	36.1	40.1
**TV viewing (hours/day)**	*124*,*441*	*4378*	*2078*	*814*
>3hours	38.1	50.7	55.5	57.7
**Meets Fruit and veg guideline** ^b^	*121*,*529*	*4275*	*2028*	*793*
NO	68.0	62.4	65.7	64.2

^a^UK Government recommendations of 150 minutes of moderate or 75 minutes of vigorous activity per week, or a combination of both

^b^5 portions of fruit/veg per day

**Fig 1:**
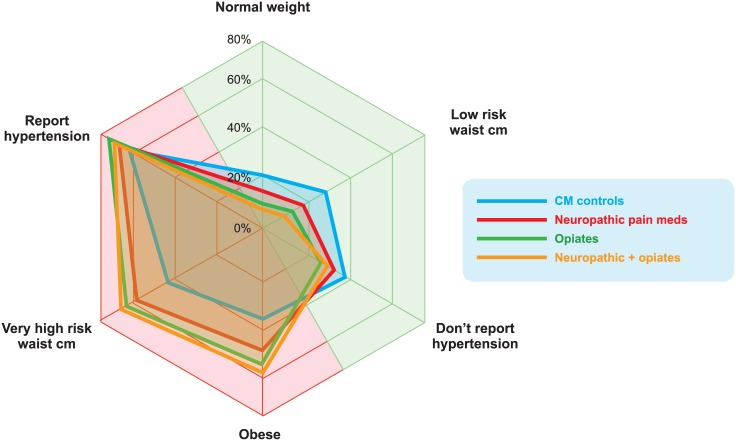
Radar chart showing the shift towards impaired cardio-metabolic health with sedative medication.

Compared with the CM controls, those taking analgesics were more likely to be obese, have a ‘very high risk’ waist cm and be diagnosed with hypertension. The proportion of obese individuals in the ‘neuropathic pain meds + opiate’ group was greater than those in the ‘CM control’ group (58.3% *vs*.35.4%, respectively). A similar pattern was observed for ‘very high risk’ waist cm, and the proportion of those with hypertension was higher across all analgesic groups (Neuropathic pain meds-67.8%, Opiates-74.5%, Neuropathic pain meds + opiates-70.7% *vs*. 62.4%). These CM characteristics are summarized in Table 2. The shift towards this poor CM health profile is visualized in Fig 1.

**Fig 2:**
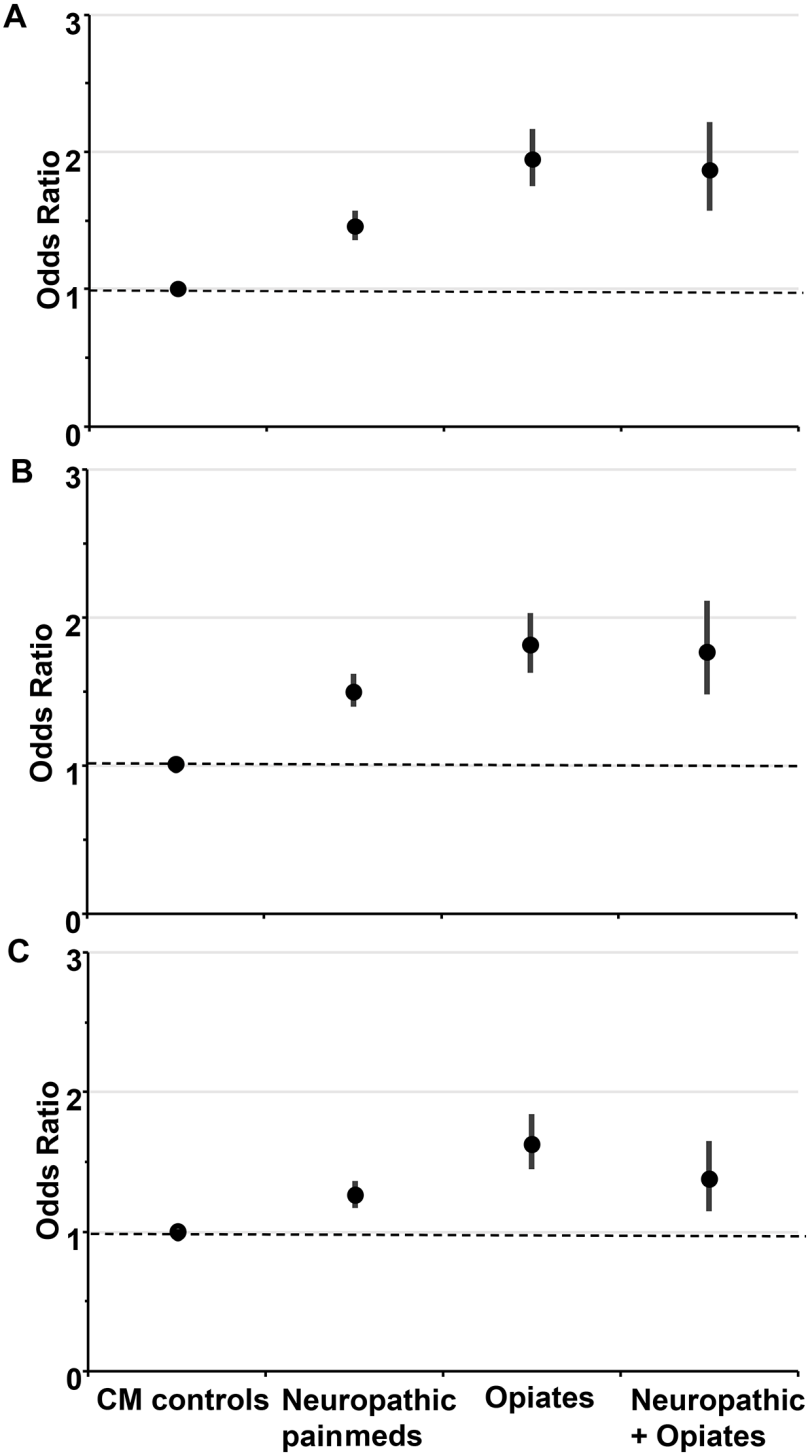
The odds of (A) being obese, (B) having a ‘very high risk’ waist cm, (C) hypertensive, according to medication group.

After controlling for sociodemographic and lifestyle factors which impact upon CM health, the odds of being obese, having a ‘very high risk’ waist cm and hypertension was significantly increased in those on analgesic medication. Those taking opiates showed the worst CM profile and were 95% more likely to be obese (OR [95%] 1.95 [1.75–2.17]), 82% more likely to have a ‘very high risk’ waist cm (1.82 [1.63–2.11]) and 63% more likely to have hypertension (1.63 [1.45–1.84]) compared to those on CM medication only. The data is shown in Table 3. The risk is visualized for medication groups in Fig 2.

**Table 3:** Odds [CI] of being obese, having a ‘very high risk’ waist cm or hypertensive according to medication group.

	Obese	Very high risk waist cm (>88 female or >102 male)	Hypertensive
**CM controls**	1.00	1.00	1.00
**Neuropathic pain meds**	1.46 [1.36–1.57]	1.50 [1.40–1.62]	1.26 [1.17–1.36]
**Opiates**	1.95 [1.75–2.17]	1.82 [1.63–2.03]	1.63 [1.45–1.84]
**Neuropathic + Opiates**	1.87 [1.57–2.22]	1.77 [1.48–2.11]	1.38 [1.15–1.65]

All models were adjusted for age, gender, ethnicity, townsend deprivation, sleep, physical activity, diet, smoking and alcohol (all models, n = 122,841).

**Fig 3:**
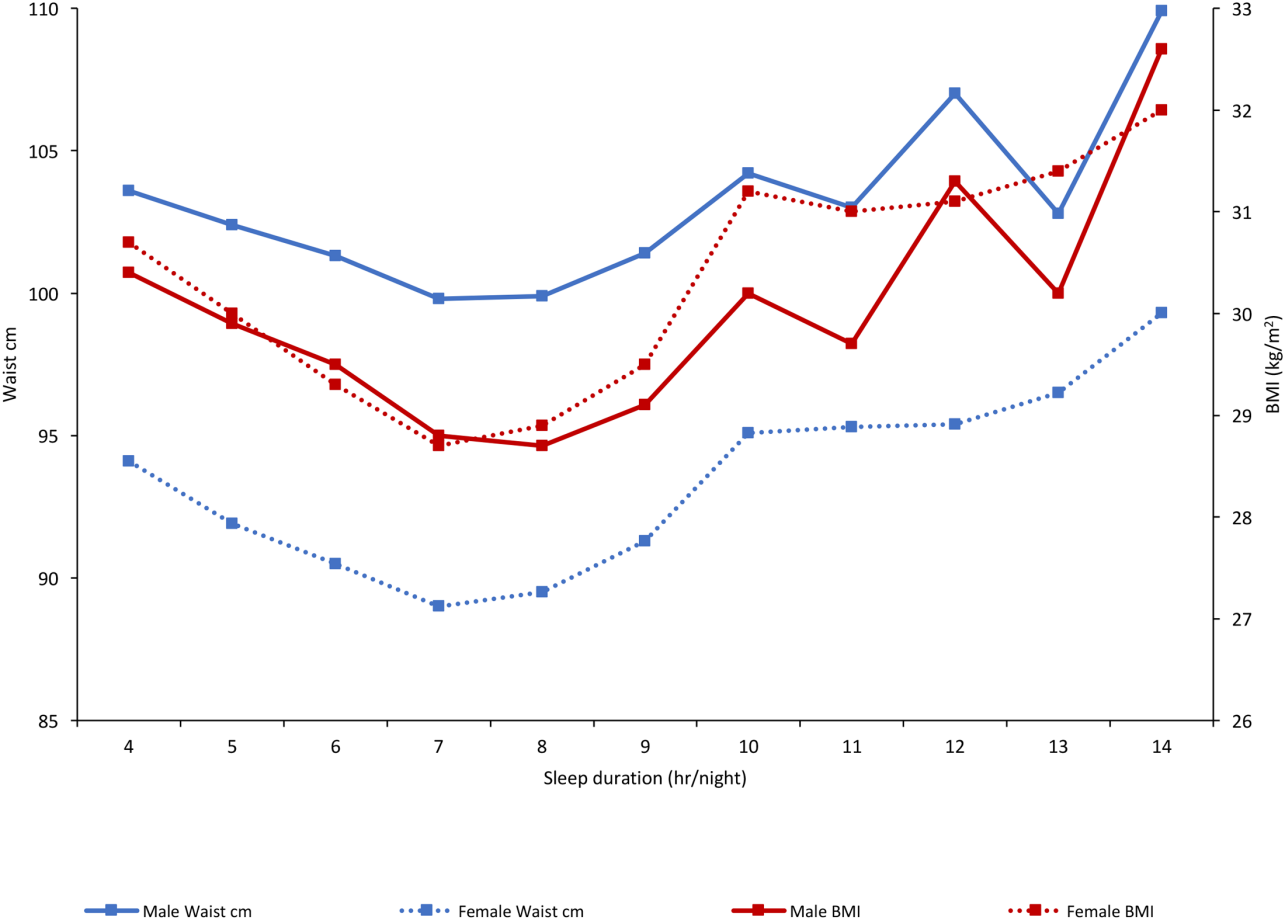
Waist cm and BMI (kg/m^2^) according to sleep duration, separated by gender (n = 133,401).

Given that analgesics are associated with sleep [24], the potential contribution to obesity from poor sleep was explored further. This expands upon work published already showing increased CM risk for those who sleep <7 hours or > 8 hours a night. Further analysis on an hour by hour basis shows a much greater risk in weight gain in those with <5 hours a night sleep or >10 hours approximating to a U shaped curve (Fig 3). However, this analysis also showed that independent of sleep duration and other risk factors, those taking analgesics were more likely to be obese, have a ‘very high risk’ waist cm and hypertension, this is summarized in Table 3.

We considered whether those with known sleep apnoea were seen more commonly in those on medication and reviewed the patient self report data for those with a diagnosis of sleep apnoea. Very few patients in any group reported a known diagnosis of sleep apnoea, only 0.6% of those on CM drugs only 1.2% of those on neuropathic pain medication, 1.5% of those on opiates, and 1.3% of those on both opiates and neuropathic pain medication.

## Discussion

This cross-sectional analysis of the UK Biobank cohort has shown that those using sedative analgesic drugs, and in particular opiates, were far more likely to have poor CM health and increased obesity compared to those on CM drugs only. Those prescribed analgesic drugs were more likely to have abnormal sleep. This is the largest study to assess the impact of analgesic drugs on CM health. It also confirms that those with poor sleep have increased obesity.

In the last two decades there has been a significant increase in the prescribing of both opioid and non-opioid medications to treat chronic pain [7]. However, this came with few trials assessing long term effects, under recognition of their potential for addiction and accidental overdose and low therapeutic ratio. Now an estimated 3–4% of Americans are prescribed opioids [8]. A recent meta-analysis showed no benefit for chronic spinal pain in those taking opiates even up to the high doses of 240mg morphine a day [25]. Opioid overdose is now the leading cause of non-injury death, quadrupling in the last 15 years [26]. However more recently, an increased rate of death outside of accidental overdose has been described. A retrospective cohort study looked at total and cause specific mortality in over 22,000 long acting opioid prescriptions and compared outcomes to those using tricyclic antidepressants or the analgesic anticonvulsants. Excluding overdose, out of hospital deaths and cardiovascular deaths were almost doubled over a six month follow up (Hazard ratio 1.72 and 1.68 respectively) with the greatest risk coming in the first 30 days of therapy [11].

In our cohort, those on opioids had far worse CM health with increased rates of hypertension and obesity. There could be a number of possible mechanisms by which opioids might be associated with weight gain. Sedation might decrease physical activity and therefore reduce energy expenditure, those in our cohort taking opiates were less active and those taking both opiates and other sedative drugs were the least active. Opioids have also been shown to alter taste perception with a craving for sugar and sweet foods described (9). Using fruit and vegetable consumption as an indicator of diet, the data suggest there was nono major difference in diet between groups in our cohort. Many taking opioids have sleep disordered breathing and disturbed sleep for any reason is associated with disruption of metabolic control (21).

Opioids are also known to worsen both snoring and untreated sleep apnoea. Only 1% of those on opiates reported a known diagnosis of sleep apnoea but this condition is known to be underdiagnosed (31) and this would appear to be the case with our cohort given an expected prevalence of at least 10% of men and 5% of women over the age of 40 [27]. Therefore, undiagnosed sleep apnoea worsened or induced by opioids could cause both impaired CM health and nocturnal hypertension.

The most widely used analgesic drugs in the antidepressant class are amitriptyline and more recently duloxetine. A number of antidepressants have been shown to effect weight and metabolism, typically via histaminergic activation [28]. Weight gain remains one of the most commonly reported adverse events and reason for discontinuation [29]. The Alpha2delta ligands pregabilin and gabapentin have been used far more frequently for chronic pain, often as an alternative to opiates but sedation and weight gain are also commonly reported adverse events with one study suggesting a dose responsive effect with 7% increase in BMI from baseline over 14 weeks [30]. Separating gabaergic drugs from amitriptyline and duloxetine (S3 Table) shows increased risks of obesity but no difference in hypertension. The mechanism of weight gain is not clear for these medications but could relate to reduction of physical activity due to sedation. To date there has not been evidence of deteriorating glycaemic control [31]. Clearly the possibility of the underlying medical condition the drug is used for being the cause of increased weight must be considered. However, weight gain is described with pregabilin, gabapentin and amitrptyline used for a wide range of causes. Given that many conditions including migraine, chronic low back pain and diabetic neuropathy are worse in those who are more obese, this raises the possibility of a viscous circle with weight gain from medication that might be effective when used acutely but when used chronically may perpetuate rather than improve pain by increasing weight.

No previous studies have reported an association between hypnotics and worse CM health and again it is not possible to determine causality but this warrants further investigation. Hypnotics have been associated with daytime sedation sufficient to impair driving and to cause falls in an older population [32], they might therefore impact on daily physical activity to contribute to obesity

The proportion of long (>9 hours/night) and short sleepers (<6 hours/night) increased in all medication groups compared to those on no medication. Fig 3 shows an association between short and long sleep and obesity (Fig 3), in agreement with previous work [33]. As many chronic non-cancer pain drugs are known to disrupt sleep, the relationship between CM health and these drugs could be driven primarily through sleep disruption. That being said, those on medication had significantly higher odds of obesity, a ‘very high risk’ waist cm and hypertension, independent of sleep duration.

Limitations of the data include a lack of knowledge about the dose of medications taken given that previous studies have suggested that higher doses of drugs cause a greater increase in BMI [12]. The additional limits of a cross sectional study is a lack of knowledge about the duration of the prescription. Further analysis of the UK Biobank cohort over time will allow a longitudinal study of the impact of those medications on long term obesity and CM health and more precise dosage analysis. The 4 groups were chosen so that the metabolic impact of drugs frequently used in combination within chronic pain could be investigated. However within the 4 groups, medications have variable modes of action, therefore we performed extra sub-group analysis with a ‘statin’ and ‘pregaballin/Gabapentin’ group due to their diverse neuromodulatory effects in the brain (S3 Table). Sleep duration was by self report only and could not distinguish causes of sleep disturbance for example undiagnosed obstructive sleep apnoea or restless legs. There may also be reporter inaccuracy. However, the findings of a U shaped curve with both very short and very long sleepers having increased mortality has been reported by a number of other large population studies, as has the association with obesity [34].

## Conclusions

This dataset has shown that those taking analgesic drugs had worse CM health and increased obesity even when controlling for multiple confounders and other commonly prescribed CM drugs. This warrants further investigation. The effect was most pronounced for opiates, and the impact upon weight, blood pressure and sleep is of concern for these DFMs. The data from this study adds further support to calls for these medications to be prescribed for shorter periods and raises further questions about the safety of their use.

## Supporting information

S1 Table List of CM drugs and their assigned values from the UK Biobank. (DOCX)

S2 Table Socio-demographics of those who have missing data for BMI, waist cm, or hypertension and therefore weren’t included in the analysis. (DOCX)

S3 Table Odds [CI] of being obese, having a ‘very high risk’ waist cm or hypertensive, according to medication group with extra sub-group analysis. (DOCX)
