# Supplementary material for: The cardio-metabolic impact of taking commonly prescribed analgesic drugs in 133,401 UK Biobank participants
Source: PLoS One. 2017 Dec 6;12(12):e0187982. doi: 10.1371/journal.pone.0187982 (PMC5718411; doi:10.1371/journal.pone.0187982)
Supplement: S1 Table — (DOCX) [file pone.0187982.s001.docx]

**S1 Table List of CM drugs and their assigned values from the UK Biobank**

| **Value** | **Drug** |
| --- | --- |
| 1140866738 | atenolol |
| 1140860806 | ramipril |
| 1141194794 | bendroflumethiazide |
| 1140916356 | losartan |
| 1140868226 | aspirin |
| 1140861806 | aspirin 75mg tablet |
| 1141168318 | clopidogrel |
| 1140861958 | simvastatin |
| 1141146234 | atorvastatin |
| 1141165470 | felodipine+ramipril |
| 1141146124 | atenolol+chlorthalidone |
| 1141146126 | atenolol+bendrofluazide |
| 1141146128 | atenolol+co-amilozide |
| 1141180778 | atenolol+chlortalidone |
| 1141194810 | atenolol+bendroflumethiazide |
| 1140860426 | atenolol+nifedipine 50mg/20mg m/r capsule |
| 1141194800 | bendroflumethiazide+potassium |
| 1141194804 | nadolol+bendroflumethiazide |
| 1141194810 | atenolol+bendroflumethiazide |
| 1141151016 | losartan potassium+hydrochlorothiazide |
| 1140864860 | nu-seals aspirin 75mg e/c tablet |
| 1141164044 | isosorbide mononitrate+aspirin |
| 1141167844 | dipyridamole+aspirin |
