# Supplementary material for: The cardio-metabolic impact of taking commonly prescribed analgesic drugs in 133,401 UK Biobank participants
Source: PLoS One. 2017 Dec 6;12(12):e0187982. doi: 10.1371/journal.pone.0187982 (PMC5718411; doi:10.1371/journal.pone.0187982)
Supplement: S2 Table — (DOCX) [file pone.0187982.s002.docx]

**S2 Table Socio-demographics of those who have missing data for BMI, waist cm, or hypertension and therefore weren’t included in the analysis**

|  | **% within each disease group** | | | |
| --- | --- | --- | --- | --- |
|  | **CM controls**  *(719)* | **Neuropathic pain meds**  *(82)* | **Opiates**  *(40)* | **Neuropathic pain meds + Opiates**  *(32)* |
| **SOCIO-DEMOGRAPHICS** | | | | |
| **% Male** | 61.3 | 61.0 | 42.5 | 59.4 |
| **Age *(n)*** | *719* | *82* | *40* | *32* |
| 37-49 | 10.4 | 13.4 | 17.5 | 25.0 |
| 50-59 | 30.9 | 36.6 | 30.0 | 31.3 |
| 60-73 | 58.7 | 50.0 | 52.5 | 43.8 |
| **Townsend deprivation quintile *(n)*** | *712* | *82* | *39* | *32* |
| 1 (least deprived) | 11.8 | 8.5 | 10.3 | 6.3 |
| 2 | 12.5 | 11.0 | 12.8 | 9.4 |
| 3 | 14.6 | 13.4 | 15.4 | 15.6 |
| 4 | 24.3 | 22.0 | 7.7 | 25.0 |
| 5 (most deprived) | 36.8 | 45.1 | 53.8 | 43.8 |
| **Ethnicity *(n)*** | *710* | *81* | *37* | *29* |
| White/British | 80.1 | 90.1 | 86.5 | 89.7 |
| Mixed | 0.8 | 1.2 | 2.7 | 0.0 |
| Asian | 11.7 | 4.9 | 5.4 | 0.0 |
| Black African | 4.4 | 3.7 | 2.7 | 6.9 |
| Chinese | 0.3 | 0.0 | 0.0 | 0.0 |
| Other | 2.7 | 0.0 | 2,7 | 3.4 |
