# Supplementary material for: The cardio-metabolic impact of taking commonly prescribed analgesic drugs in 133,401 UK Biobank participants
Source: PLoS One. 2017 Dec 6;12(12):e0187982. doi: 10.1371/journal.pone.0187982 (PMC5718411; doi:10.1371/journal.pone.0187982)
Supplement: S3 Table — (DOCX) [file pone.0187982.s003.docx]

**S3 Table. Odds [CI] of being obese, having a ‘very high risk’ waist cm or hypertensive, according to medication group with extra sub-group analysis.**

|  | **Obese** | **Very high risk waist cm (>88 female or >102 male)** | **Hypertensive** |
| --- | --- | --- | --- |
| **CM controls** | 1.00 | 1.00 | 1.00 |
| **Statins** | 1.21 [1.24-1.76] | 1.25 [1.22-1.28] | 0.80 [0.78-0.83] |
| **Neuropathic pain meds** | 1.48 [1.36-1.61] | 1.55 [1.42-1.69] | 1.14 [1.04-1.25] |
| **Pregabilin/**  **Gabapentin** | 2.00 [1.75-2.30] | 2.12 [1.85-2.44] | 1.07 [0.93-1.23]* |
| **Opiates** | 2.16 [1.94-2.41] | 2.06 [1.84-2.30] | 1.45 [1.29-1.63] |
| **Neuropathic + Opiates** | 2.03 [1.71-2.41] | 2.01 [1.69-2.41] | 1.22 [1.01-1.46] |

However within the 4 groups, medications have variable modes of action, therefore we performed extra sub-group analysis with a ‘statin’ and ‘pregaballin/Gabapentin’ group due to their diverse neuromodulatory effects in the brain
